# Supplementary material for: Pyruvate Dehydrogenase Kinase 1 inhibition mediated oxidative phosphorylation enhancement in cartilage promotes osteoarthritis progression
Source: BMC Musculoskelet Disord. 2023 Jul 20;24:597. doi: 10.1186/s12891-023-06585-6 (PMC10357736; doi:10.1186/s12891-023-06585-6)
Supplement: Supplementary file 1 — Additional file 1. [file 12891_2023_6585_MOESM1_ESM.zip › MF.Gsea.1653627316242/GOMF_PROTON_TRANSMEMBRANE_TRANSPORTER_ACTIVITY.html]

Details for gene set GOMF\_PROTON\_TRANSMEMBRANE\_TRANSPORTER\_ACTIVITY[GSEA]

|  || Dataset | 55457\_collapsed\_to\_symbols.cls.cls #OA\_versus\_normal.cls.cls #OA\_versus\_normal\_repos |
| Phenotype | cls.cls#OA\_versus\_normal\_repos |
| Upregulated in class | OA |
| GeneSet | GOMF\_PROTON\_TRANSMEMBRANE\_TRANSPORTER\_ACTIVITY |
| Enrichment Score (ES) | 0.51817966 |
| Normalized Enrichment Score (NES) | 2.375416 |
| Nominal p-value | 0.0 |
| FDR q-value | 0.0 |
| FWER p-Value | 0.0 |
Table: GSEA Results Summary

  

Fig 1: Enrichment plot: GOMF\_PROTON\_TRANSMEMBRANE\_TRANSPORTER\_ACTIVITY      
 Profile of the Running ES Score & Positions of GeneSet Members on the Rank Ordered List

  

| SYMBOL | TITLE | RANK IN GENE LIST | RANK METRIC SCORE | RUNNING ES | CORE ENRICHMENT || 1 | TMCO3 | transmembrane and coiled-coil domains 3 [Source:HGNC Symbol;Acc:HGNC:20329] | 12 | 1.165 | 0.0375 | Yes |
| 2 | ATP6V1C1 | ATPase H+ transporting V1 subunit C1 [Source:HGNC Symbol;Acc:HGNC:856] | 17 | 1.125 | 0.0744 | Yes |
| 3 | UQCR10 | "ubiquinol-cytochrome c reductase, complex III subunit X [Source:HGNC Symbol;Acc:HGNC:30863]" | 53 | 0.987 | 0.1042 | Yes |
| 4 | ATP6V0D1 | ATPase H+ transporting V0 subunit d1 [Source:HGNC Symbol;Acc:HGNC:13724] | 135 | 0.872 | 0.1267 | Yes |
| 5 | SLC22A18 | solute carrier family 22 member 18 [Source:HGNC Symbol;Acc:HGNC:10964] | 152 | 0.858 | 0.1538 | Yes |
| 6 | ATP6V1E1 | ATPase H+ transporting V1 subunit E1 [Source:HGNC Symbol;Acc:HGNC:857] | 287 | 0.758 | 0.1684 | Yes |
| 7 | ATP6V0B | ATPase H+ transporting V0 subunit b [Source:HGNC Symbol;Acc:HGNC:861] | 382 | 0.713 | 0.1847 | Yes |
| 8 | ATP5MC1 | ATP synthase membrane subunit c locus 1 [Source:HGNC Symbol;Acc:HGNC:841] | 465 | 0.674 | 0.2005 | Yes |
| 9 | UQCRC1 | ubiquinol-cytochrome c reductase core protein 1 [Source:HGNC Symbol;Acc:HGNC:12585] | 473 | 0.672 | 0.2222 | Yes |
| 10 | ATP6V1G1 | ATPase H+ transporting V1 subunit G1 [Source:HGNC Symbol;Acc:HGNC:864] | 515 | 0.655 | 0.2406 | Yes |
| 11 | ATP6V0E2 | ATPase H+ transporting V0 subunit e2 [Source:HGNC Symbol;Acc:HGNC:21723] | 532 | 0.647 | 0.2607 | Yes |
| 12 | ATP5PD | ATP synthase peripheral stalk subunit d [Source:HGNC Symbol;Acc:HGNC:845] | 564 | 0.635 | 0.2793 | Yes |
| 13 | COX5B | cytochrome c oxidase subunit 5B [Source:HGNC Symbol;Acc:HGNC:2269] | 585 | 0.628 | 0.2985 | Yes |
| 14 | COX6A1 | cytochrome c oxidase subunit 6A1 [Source:HGNC Symbol;Acc:HGNC:2277] | 653 | 0.605 | 0.3132 | Yes |
| 15 | DMAC2L | distal membrane arm assembly component 2 like [Source:HGNC Symbol;Acc:HGNC:18799] | 695 | 0.595 | 0.3297 | Yes |
| 16 | ATP6V1D | ATPase H+ transporting V1 subunit D [Source:HGNC Symbol;Acc:HGNC:13527] | 750 | 0.581 | 0.3446 | Yes |
| 17 | ATP5PO | ATP synthase peripheral stalk subunit OSCP [Source:HGNC Symbol;Acc:HGNC:850] | 840 | 0.558 | 0.3561 | Yes |
| 18 | SLC2A10 | solute carrier family 2 member 10 [Source:HGNC Symbol;Acc:HGNC:13444] | 874 | 0.550 | 0.3717 | Yes |
| 19 | SLC9A6 | solute carrier family 9 member A6 [Source:HGNC Symbol;Acc:HGNC:11079] | 981 | 0.522 | 0.3807 | Yes |
| 20 | COX8A | cytochrome c oxidase subunit 8A [Source:HGNC Symbol;Acc:HGNC:2294] | 1105 | 0.494 | 0.3874 | Yes |
| 21 | ATP6V0E1 | ATPase H+ transporting V0 subunit e1 [Source:HGNC Symbol;Acc:HGNC:863] | 1207 | 0.474 | 0.3952 | Yes |
| 22 | ATP5PB | ATP synthase peripheral stalk-membrane subunit b [Source:HGNC Symbol;Acc:HGNC:840] | 1293 | 0.459 | 0.4038 | Yes |
| 23 | ATP6V1H | ATPase H+ transporting V1 subunit H [Source:HGNC Symbol;Acc:HGNC:18303] | 1304 | 0.457 | 0.4181 | Yes |
| 24 | ATP5MF | ATP synthase membrane subunit f [Source:HGNC Symbol;Acc:HGNC:848] | 1330 | 0.453 | 0.4311 | Yes |
| 25 | CLCN3 | chloride voltage-gated channel 3 [Source:HGNC Symbol;Acc:HGNC:2021] | 1535 | 0.423 | 0.4291 | Yes |
| 26 | SLC2A9 | solute carrier family 2 member 9 [Source:HGNC Symbol;Acc:HGNC:13446] | 1634 | 0.408 | 0.4350 | Yes |
| 27 | SURF1 | SURF1 cytochrome c oxidase assembly factor [Source:HGNC Symbol;Acc:HGNC:11474] | 1829 | 0.381 | 0.4324 | Yes |
| 28 | COX7A1 | cytochrome c oxidase subunit 7A1 [Source:HGNC Symbol;Acc:HGNC:2287] | 2045 | 0.356 | 0.4274 | Yes |
| 29 | SLC9A3 | solute carrier family 9 member A3 [Source:HGNC Symbol;Acc:HGNC:11073] | 2148 | 0.344 | 0.4308 | Yes |
| 30 | COX4I1 | cytochrome c oxidase subunit 4I1 [Source:HGNC Symbol;Acc:HGNC:2265] | 2167 | 0.342 | 0.4407 | Yes |
| 31 | ATP5F1C | ATP synthase F1 subunit gamma [Source:HGNC Symbol;Acc:HGNC:833] | 2190 | 0.339 | 0.4502 | Yes |
| 32 | ATP12A | ATPase H+/K+ transporting non-gastric alpha2 subunit [Source:HGNC Symbol;Acc:HGNC:13816] | 2330 | 0.323 | 0.4500 | Yes |
| 33 | ATP5MC2 | ATP synthase membrane subunit c locus 2 [Source:HGNC Symbol;Acc:HGNC:842] | 2371 | 0.318 | 0.4574 | Yes |
| 34 | NNT | nicotinamide nucleotide transhydrogenase [Source:HGNC Symbol;Acc:HGNC:7863] | 2425 | 0.311 | 0.4635 | Yes |
| 35 | CLCN4 | chloride voltage-gated channel 4 [Source:HGNC Symbol;Acc:HGNC:2022] | 2498 | 0.303 | 0.4679 | Yes |
| 36 | COX7A2L | cytochrome c oxidase subunit 7A2 like [Source:HGNC Symbol;Acc:HGNC:2289] | 2517 | 0.302 | 0.4765 | Yes |
| 37 | SLC15A3 | solute carrier family 15 member 3 [Source:HGNC Symbol;Acc:HGNC:18068] | 2612 | 0.290 | 0.4787 | Yes |
| 38 | ATP5MC3 | ATP synthase membrane subunit c locus 3 [Source:HGNC Symbol;Acc:HGNC:843] | 2643 | 0.286 | 0.4858 | Yes |
| 39 | SLC17A5 | solute carrier family 17 member 5 [Source:HGNC Symbol;Acc:HGNC:10933] | 2671 | 0.283 | 0.4930 | Yes |
| 40 | ATP5F1D | ATP synthase F1 subunit delta [Source:HGNC Symbol;Acc:HGNC:837] | 2701 | 0.279 | 0.5000 | Yes |
| 41 | ATP6V0C | ATPase H+ transporting V0 subunit c [Source:HGNC Symbol;Acc:HGNC:855] | 2790 | 0.271 | 0.5021 | Yes |
| 42 | SLC9A7 | solute carrier family 9 member A7 [Source:HGNC Symbol;Acc:HGNC:17123] | 2817 | 0.268 | 0.5089 | Yes |
| 43 | COX6B1 | cytochrome c oxidase subunit 6B1 [Source:HGNC Symbol;Acc:HGNC:2280] | 2915 | 0.259 | 0.5099 | Yes |
| 44 | ATP5MG | ATP synthase membrane subunit g [Source:HGNC Symbol;Acc:HGNC:14247] | 2941 | 0.256 | 0.5164 | Yes |
| 45 | ATP5F1B | ATP synthase F1 subunit beta [Source:HGNC Symbol;Acc:HGNC:830] | 3024 | 0.247 | 0.5182 | Yes |
| 46 | ATP4B | ATPase H+/K+ transporting subunit beta [Source:HGNC Symbol;Acc:HGNC:820] | 3160 | 0.234 | 0.5154 | No |
| 47 | SLC33A1 | solute carrier family 33 member 1 [Source:HGNC Symbol;Acc:HGNC:95] | 3320 | 0.219 | 0.5102 | No |
| 48 | CTNS | "cystinosin, lysosomal cystine transporter [Source:HGNC Symbol;Acc:HGNC:2518]" | 3337 | 0.218 | 0.5162 | No |
| 49 | COX7B | cytochrome c oxidase subunit 7B [Source:HGNC Symbol;Acc:HGNC:2291] | 3452 | 0.207 | 0.5141 | No |
| 50 | COX15 | cytochrome c oxidase assembly homolog COX15 [Source:HGNC Symbol;Acc:HGNC:2263] | 3727 | 0.183 | 0.4987 | No |
| 51 | UQCRH | ubiquinol-cytochrome c reductase hinge protein [Source:HGNC Symbol;Acc:HGNC:12590] | 3827 | 0.174 | 0.4968 | No |
| 52 | ATP6V1F | ATPase H+ transporting V1 subunit F [Source:HGNC Symbol;Acc:HGNC:16832] | 3833 | 0.173 | 0.5021 | No |
| 53 | ATP5F1A | ATP synthase F1 subunit alpha [Source:HGNC Symbol;Acc:HGNC:823] | 4137 | 0.143 | 0.4831 | No |
| 54 | SLC9A1 | solute carrier family 9 member A1 [Source:HGNC Symbol;Acc:HGNC:11071] | 4221 | 0.135 | 0.4811 | No |
| 55 | NOX5 | NADPH oxidase 5 [Source:HGNC Symbol;Acc:HGNC:14874] | 4263 | 0.131 | 0.4823 | No |
| 56 | ATP6V0A1 | ATPase H+ transporting V0 subunit a1 [Source:HGNC Symbol;Acc:HGNC:865] | 4371 | 0.123 | 0.4780 | No |
| 57 | CLCN5 | chloride voltage-gated channel 5 [Source:HGNC Symbol;Acc:HGNC:2023] | 4372 | 0.123 | 0.4820 | No |
| 58 | CYC1 | cytochrome c1 [Source:HGNC Symbol;Acc:HGNC:2579] | 4879 | 0.082 | 0.4452 | No |
| 59 | SLC9A2 | solute carrier family 9 member A2 [Source:HGNC Symbol;Acc:HGNC:11072] | 5068 | 0.067 | 0.4328 | No |
| 60 | SLC25A4 | solute carrier family 25 member 4 [Source:HGNC Symbol;Acc:HGNC:10990] | 5073 | 0.067 | 0.4347 | No |
| 61 | COX6A2 | cytochrome c oxidase subunit 6A2 [Source:HGNC Symbol;Acc:HGNC:2279] | 5242 | 0.053 | 0.4233 | No |
| 62 | COX5A | cytochrome c oxidase subunit 5A [Source:HGNC Symbol;Acc:HGNC:2267] | 5329 | 0.045 | 0.4181 | No |
| 63 | UQCRFS1 | "ubiquinol-cytochrome c reductase, Rieske iron-sulfur polypeptide 1 [Source:HGNC Symbol;Acc:HGNC:12587]" | 5509 | 0.032 | 0.4052 | No |
| 64 | CYB5A | cytochrome b5 type A [Source:HGNC Symbol;Acc:HGNC:2570] | 5850 | 0.005 | 0.3788 | No |
| 65 | ATP6V0A4 | ATPase H+ transporting V0 subunit a4 [Source:HGNC Symbol;Acc:HGNC:866] | 5968 | -0.002 | 0.3698 | No |
| 66 | ATP5PF | ATP synthase peripheral stalk subunit F6 [Source:HGNC Symbol;Acc:HGNC:847] | 6109 | -0.013 | 0.3593 | No |
| 67 | ATP5ME | ATP synthase membrane subunit e [Source:HGNC Symbol;Acc:HGNC:846] | 6365 | -0.032 | 0.3404 | No |
| 68 | ATP5F1E | ATP synthase F1 subunit epsilon [Source:HGNC Symbol;Acc:HGNC:838] | 6405 | -0.036 | 0.3386 | No |
| 69 | SLC39A8 | solute carrier family 39 member 8 [Source:HGNC Symbol;Acc:HGNC:20862] | 6450 | -0.039 | 0.3364 | No |
| 70 | SLC15A1 | solute carrier family 15 member 1 [Source:HGNC Symbol;Acc:HGNC:10920] | 6515 | -0.043 | 0.3328 | No |
| 71 | SLC15A2 | solute carrier family 15 member 2 [Source:HGNC Symbol;Acc:HGNC:10921] | 6704 | -0.056 | 0.3200 | No |
| 72 | ATP6V1B2 | ATPase H+ transporting V1 subunit B2 [Source:HGNC Symbol;Acc:HGNC:854] | 7074 | -0.086 | 0.2941 | No |
| 73 | SLC45A2 | solute carrier family 45 member 2 [Source:HGNC Symbol;Acc:HGNC:16472] | 7144 | -0.092 | 0.2917 | No |
| 74 | SLC11A1 | solute carrier family 11 member 1 [Source:HGNC Symbol;Acc:HGNC:10907] | 7153 | -0.092 | 0.2941 | No |
| 75 | TCIRG1 | "T cell immune regulator 1, ATPase H+ transporting V0 subunit a3 [Source:HGNC Symbol;Acc:HGNC:11647]" | 7600 | -0.127 | 0.2635 | No |
| 76 | SLC36A1 | solute carrier family 36 member 1 [Source:HGNC Symbol;Acc:HGNC:18761] | 7670 | -0.132 | 0.2625 | No |
| 77 | SLC25A5 | solute carrier family 25 member 5 [Source:HGNC Symbol;Acc:HGNC:10991] | 7877 | -0.146 | 0.2513 | No |
| 78 | ATP6V1A | ATPase H+ transporting V1 subunit A [Source:HGNC Symbol;Acc:HGNC:851] | 8013 | -0.156 | 0.2459 | No |
| 79 | SLC25A3 | solute carrier family 25 member 3 [Source:HGNC Symbol;Acc:HGNC:10989] | 8681 | -0.208 | 0.2007 | No |
| 80 | SLC25A22 | solute carrier family 25 member 22 [Source:HGNC Symbol;Acc:HGNC:19954] | 8682 | -0.208 | 0.2075 | No |
| 81 | ATP6V0A2 | ATPase H+ transporting V0 subunit a2 [Source:HGNC Symbol;Acc:HGNC:18481] | 8843 | -0.221 | 0.2023 | No |
| 82 | SLC11A2 | solute carrier family 11 member 2 [Source:HGNC Symbol;Acc:HGNC:10908] | 9152 | -0.244 | 0.1864 | No |
| 83 | SLC9A8 | solute carrier family 9 member A8 [Source:HGNC Symbol;Acc:HGNC:20728] | 10170 | -0.338 | 0.1182 | No |
| 84 | ATP4A | ATPase H+/K+ transporting subunit alpha [Source:HGNC Symbol;Acc:HGNC:819] | 10417 | -0.363 | 0.1109 | No |
| 85 | SLC47A1 | solute carrier family 47 member 1 [Source:HGNC Symbol;Acc:HGNC:25588] | 10423 | -0.363 | 0.1225 | No |
| 86 | ATP6V1B1 | ATPase H+ transporting V1 subunit B1 [Source:HGNC Symbol;Acc:HGNC:853] | 12206 | -0.646 | 0.0048 | No |
| 87 | LETM1 | leucine zipper and EF-hand containing transmembrane protein 1 [Source:HGNC Symbol;Acc:HGNC:6556] | 12272 | -0.669 | 0.0218 | No |
| 88 | SLC9A5 | solute carrier family 9 member A5 [Source:HGNC Symbol;Acc:HGNC:11078] | 12642 | -0.820 | 0.0201 | No |
Table: GSEA details [plain text format]

  

Fig 2: GOMF\_PROTON\_TRANSMEMBRANE\_TRANSPORTER\_ACTIVITY      
 Blue-Pink O' Gram in the Space of the Analyzed GeneSet

  

Fig 3: GOMF\_PROTON\_TRANSMEMBRANE\_TRANSPORTER\_ACTIVITY: Random ES distribution      
 Gene set null distribution of ES for **GOMF\_PROTON\_TRANSMEMBRANE\_TRANSPORTER\_ACTIVITY**
